# Fractionating the Neurocognitive Mechanisms Underlying Working Memory: Independent Effects of Dopamine and Parkinson’s Disease

**DOI:** 10.1093/cercor/bhx242

**Published:** 2017-10-13

**Authors:** Sean James Fallon, Rozemarijn Margaretha Mattiesing, Kinan Muhammed, Sanjay Manohar, Masud Husain

**Affiliations:** 1 Department of Experimental Psychology, University of Oxford, New Radcliffe House, Walton Street, Oxford, OX2 6AG, UK; 2 Department of Clinical Neurosciences, John Radcliffe Hospital, Oxford, OX3 9DU, UK

**Keywords:** Cognitive control, dopamine, Parkinson’s disease, working memory

## Abstract

Deficits in working memory (WM) in Parkinson’s disease (PD) are often considered to be secondary to dopaminergic depletion. However, the neurocognitive mechanisms by which dopamine causes these deficits remain highly contested, and PD is now also known to be associated with nondopaminergic pathology. Here, we examined how PD and dopaminergic medication modulate three components of WM: maintenance over time, updating contents with new information and making memories distracter-resistant. Compared with controls, patients were disproportionately impaired when retaining information for longer durations. By applying a probabilistic model, we were able to reveal that the source of this error was selectively due to precision of memory representations degrading over time. By contrast, replenishing dopamine levels in PD improved executive control over both the ability to ignore and update, but did not affect maintenance of information across time. This was due to a decrease in guess responses, consistent with the view that dopamine serves to prevent WM representations being corrupted by irrelevant information, but has no impact on information decay. Cumulatively, these results reveal a dissociation in the neural mechanisms underlying poor WM: whereas dopamine reduces interference, nondopaminergic systems in PD appear to modulate processes that prevent information decaying more quickly over time.

## Introduction

Our understanding of the importance of dopamine in human brain function has been influenced greatly by observations on Parkinson’s disease (PD). As well as the conspicuous motor deficits associated with the condition, cognitive impairment is now well-established as a major feature (Kehagia et al. 2012; Gratwicke et al. 2015). Working memory (WM), the means by which information is retained and manipulated over short intervals, has been found to be particularly impaired in PD (Lewis et al. 2003, 2005; Sawamoto et al. 2008). Yet, our ability to effectively use PD as a model of dopamine depletion and to improve treatments for the associated cognitive symptoms has been hampered by a lack of understanding about the precise contribution of dopamine to patients’ cognitive impairment. While dopaminergic depletion has intuitively been considered to be the prime suspect underlying mnemonic impairments, this is not necessarily the case. PD is also associated with other neurochemical disturbances to the serotonergic, noradrenergic and cholinergic systems (Dubois et al. 1983; Zarow et al. 2003; Kish et al. 2008).

One of the ways in which investigators can potentially attempt to isolate the contribution of dopamine is to test patients ON and OFF their dopaminergic medication (Poewe et al. 1991; Lange et al. 1992; Lewis et al. 2005; Hughes et al. 2013). This approach has the benefit of ensuring that each patient acts as their own control, and crucially any nondopaminergic pathology or effects should remain constant across ON and OFF sessions. However, under such circumstances, the contribution of dopamine to WM impairments has been subject to conflicting results and rival hypotheses. For example, dopaminergic medication has been reported both to improve WM (Lange et al. 1992) and impair performance in patients (Poewe et al. 1991; Cools et al. 2010; Uitvlugt et al. 2016), though note that the pattern of results may also vary by modality (Owen et al. 1997; Postle et al. 1997; Gruszka et al. 2016). One potential explanation for these competing findings is that maintaining information in memory might actually be intact in PD, but manipulation is impaired (Lewis et al. 2005). However, progress in this area has been hampered by the fact that there is a surprising lack of understanding of the separate consequences of maintaining and manipulating information in WM, not only in PD but also in healthy people.

In studies of healthy individuals, there has been a long debate regarding the mechanisms underlying forgetting over the short-term (Portrat et al. 2008; Baddeley 2012; Oberauer et al. 2012). Some argue that information is degraded through temporal decay, with longer retention periods leading to erosion of the fidelity of mental representations (Hitch et al. 2001). In the alternative view, the predominant cause of forgetting is interference between items in WM and processing of distracters (Lewandowsky et al. 2010). Some investigators have even presented evidence for the influence of both factors in healthy people (Pertzov et al. 2016). In PD, too, it is unclear how much forgetting over the short-term is due to decay or interference. If maintenance of information is intact in PD, there should be no appreciable difference from healthy controls for pure decay across time. In contrast, if manipulation of information—such as updating the contents of WM—causes impaired recall in PD then interference might be the crucial factor.

Updating is not the only process involved in choreographing the control of relevant versus irrelevant items into WM. Another key process is active protection of memoranda from irrelevant information—the ability to ignore distracting items. It has been proposed that updating and ignoring, two key processes of dealing with irrelevant information, might lean on distinct, separate fronto-striatal systems (McNab and Klingberg 2008; Baier et al. 2010; Yu et al. 2013; Fallon and Cools 2014; Ekman et al. 2016). Moreover, dopamine has been hypothesized to have a causal role in modulating the gating function that either allows new information to enter WM (to update it), or protects the contents of WM by keeping the gate shut (to ignore distracters effectively). Thus, it has been proposed that dopamine levels in the striatum (Hazy et al. 2007) or frontal cortex (Durstewitz and Seamans 2008) play an important role in promoting a shift in the balance between opening or closing the gate. In support of this view, methylphenidate, a drug that enhances both dopamine and noradrenaline levels, can simultaneously improve ignoring but impair updating (Fallon et al. 2016a).

Notwithstanding these interesting findings, there remains uncertainty over whether updating and ignoring are indeed orthogonal tasks (gate open or shut) in the normal healthy brain, especially when not perturbed by either drug or rewards (Fallon and Cools 2014). Furthermore, the independent effects of ignoring, updating and simple maintenance over time have not been established within the same paradigm. Moreover, an alternative conceptualization of ignoring and updating is that they both involve dealing with irrelevant information—albeit the former proactively and the latter retroactively. Thus, if dopamine is responsible for being the arbiter of the lifespan of irrelevant information in the brain, then ignoring and updating may be similarly affected patients' medication. In this study we sought to overcome these issues by using principles from a relatively new type of WM paradigm that measures quality of recall (Ma et al. 2014; Fallon et al. 2016b).

**Figure 1.**
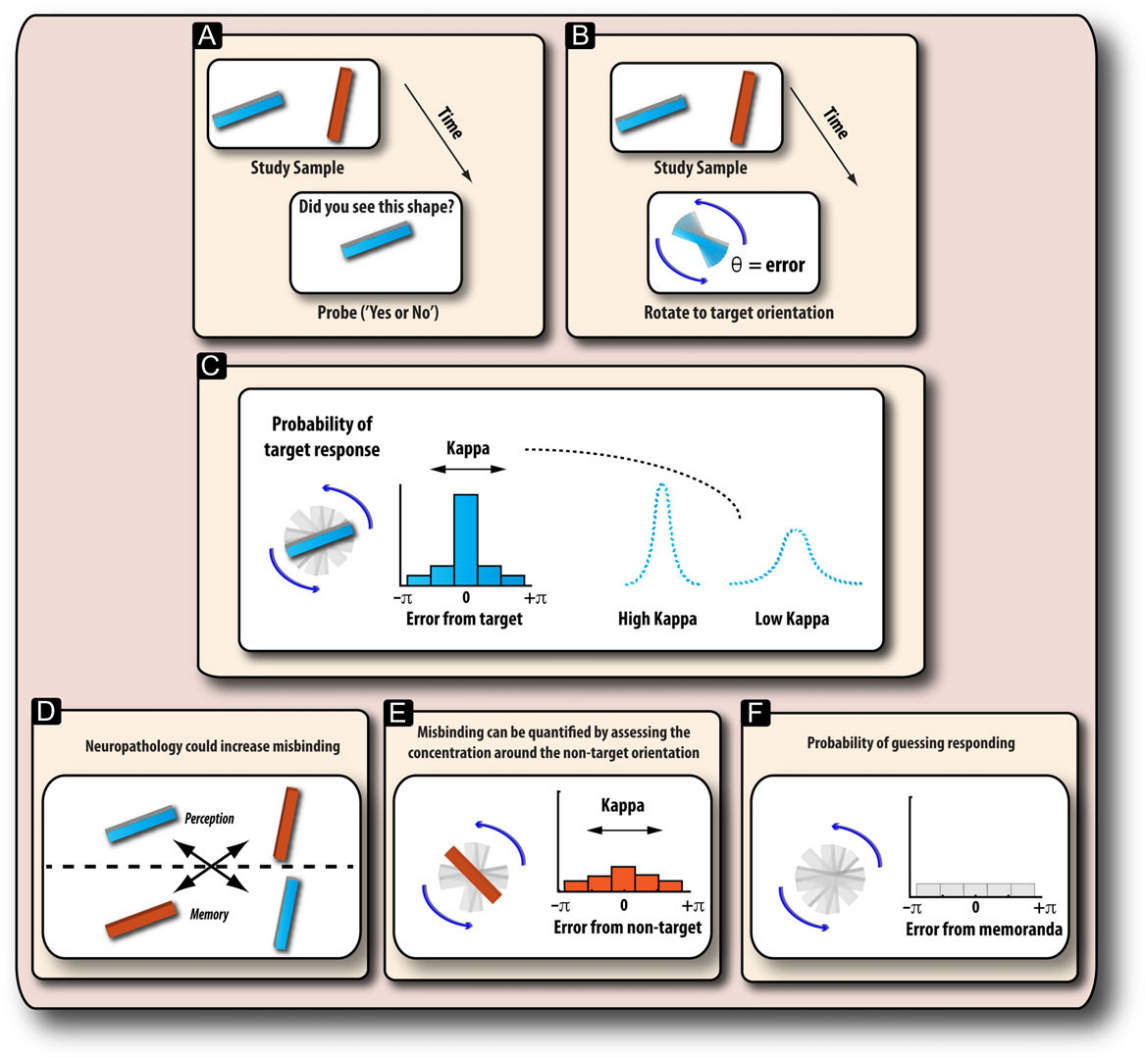
Measuring recall on WM tasks. (*A*) Traditional studies on WM in PD have used binary match-to-sample tasks, which dichotomizes recall of memoranda (Did you remember it or not?). (*B*) An alternative method asks participants to reproduce the exact feature of a previously seen item, for example, its orientation, using a continuous, analog—not binary—response space. (*C*) By applying a computational model of WM (Bays et al., 2009), the source of recall errors on WM tasks can be deconstructed. Error can result from a change in the variability of remembering memoranda. This is captured in the model in terms of the parameter [kappa]. Higher kappa values indicate lower variability in the response of retained items (for both targets or nontargets). (*D*) When remembering several items, it is important not only to remember their orientation, but also to correctly bind, or associate, an orientation with a specific color. (*E*) Misbinding errors occur when an orientation is erroneously associated with the wrong color. For example, if the orange bar appeared at an orientation of 10° and the blue bar at 75°, a misbinding error occurs if a participant rotates the probed orange bar (target) to the remembered orientation of the blue bar. (*F*) Error can also occur due to random guesses which might be due to the representation of an item disappearing completely, so the response bears no relation to the orientations that were actually presented, i.e., they are flat with respect to the orientation of the targets or nontargets displayed in the memory array.

Traditional measures of WM have usually relied on examining the quantity of information—the number of items—that can be correctly remembered and have indexed recall in a binary fashion (correct or incorrect response; Fig. 1*A*). However, more recent studies have interrogated the precision with which participants can reproduce the exact features of visual memoranda, such as their orientation, location, or color (Wilken and Ma 2004; Gorgoraptis et al. 2011; Pertzov et al. 2012) (Fig. 1*B*). Such methods require participants to reproduce a feature of a remembered item such as orientation, using a continuous, analog response space, not a binary (correct or incorrect) response. Thus they provide a graded measure of an item's fidelity in WM and, moreover, have been shown to provide enhanced sensitivity to detect changes in PD patients' recall (Zokaei et al. 2015).

In addition, the results from this type of delayed reproduction task have been analyzed using a computational model of WM that can uncover the latent structure of mental representations from the pattern of participants’ responses. In this model, WM recall is considered to result from a combination of factors: the precision (fidelity) with which an item is maintained (Fig. 1*C*); the probability of responding to the target; the likelihood of responding to nontargets presented; and finally, the probability of guessing randomly (Fig. 1*D***–***F*).

In this study, we exploit these methodological advances to examine the relationship between ignoring and updating performance in patients compared to controls, and how patients' performance varies according to their dopaminergic state. A key methodological issue that has not satisfactorily been addressed previously is ensuring that the processes of maintaining and manipulating information are properly matched for the temporal duration over which information needs to be retained. In many studies of WM manipulation, (e.g., Lewis et al. 2005), a secondary task such as updating the contents of memory, is inserted during a constant retention interval, but this inevitably means that the duration over which items are maintained in the update and control conditions are unequal. The updated items are at an advantage because they enter WM more recently than items stored in the control condition where no updating is required.

Here, by controlling for duration of retention in different types of manipulation trials versus simple maintenance, we are able to assess the role of dopamine on three different WM processes—maintaining, ignoring, and updating. In theory, by fractionating processes involved in manipulation of irrelevant items in WM and by controlling for the temporal duration over which information is maintained, the distinct contribution dopamine has on patients’ mnemonic impairment might be isolated by examining performance ON and OFF dopamine. Conversely, the nondopaminergic deficits present in PD should appear refractory to changes in medication state allowing them also to be identified.

We compared PD patients to elderly healthy controls and patients tested ON and OFF their dopaminergic medication to examine the extent to which dopamine differentially affected maintenance versus manipulation of items in WM and whether, in line with models of WM gating (Hazy et al. 2007; Durstewitz and Seamans 2008), dopamine had opposite effects on ignoring and updating.

## Methods

### Participants

This study consisted of 2 separate samples: healthy older adults and patients with PD. Twenty patients with idiopathic PD fulfilling the criteria of the UK Parkinson’s disease Society Brain Bank (Gibb and Lees 1988) and without dementia participated. Patients were eligible to take part if they were currently on dopaminergic medication and had no history of any other major neurological or psychiatric illnesses. Seventeen healthy older adults were also recruited. Patient and control demographics are presented in Supplementary Table 1. One Addenbrooke’s Cognitive Examination (ACE) score from the patient group was missing, but that patient’s Montreal Cognitive Assessment (MOCA) score (30/30) showed good performance. To be eligible for inclusion, all participants had to have normal or corrected-to-normal vision and were not color blind. They gave informed written consent prior to participation. Data from 1 patient were excluded because for one session they professed to suffering immense difficulty concentrating on the task. Their exclusion did not affect the pattern or significance of results. To examine the effect of the level of medication in influencing our results we calculated equivalent levodopa dose (Tomlinson et al. 2010).

### Design

**Figure 2.**
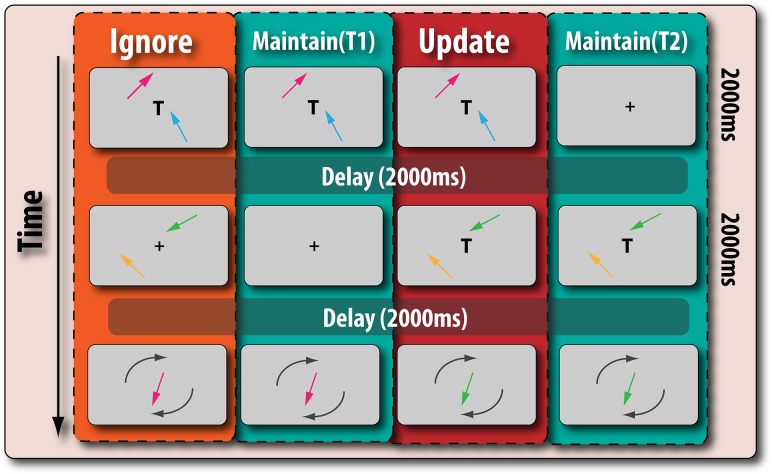
Two colored arrows with different orientations were presented for 2 s. In all 4 conditions, the resolution, or fidelity, of their recall was probed at the end with a colored arrow presented at screen center, which participants had to rotate so that it matched the target orientation. The trial types differed according to whether irrelevant information was presented and the time for which memoranda had to be retained. The trials were randomly intermixed. Participants were instructed to remember only the most recently presented pair of arrows that were shown with the letter “T”, which designated the potential target arrows. In the ignore condition (Far left), participants had to retain information whilst ignoring an irrelevant pair of arrows presented during maintenance—no “T” was presented when these distracters were shown. In contrast, in the update condition, participants were presented with 2 pairs of arrows consecutively, both of which were presented with the letter “T”. They had to remember the last pair of arrows, and discard the previous pair of arrows, which were now irrelevant. Two control conditions did not feature any irrelevant material but differed only in the length of time for which the information had to be retained. The maintain (T1) condition served as the temporal control for the ignore condition, whereas the maintain (T2) condition served as the temporal control for the update condition.

Participants performed a delayed reproduction (or adjustment) task that allowed the fidelity of WM representations to be assessed. Such paradigms share the common feature that participants are shown memoranda that vary in a parametric manner e.g., their orientation, and have to reproduce this feature as best as they can. Here, we examined the ability of participants to encode the orientation of a pair of arrows and then assess the fidelity with which one of these orientations could be reproduced (Fig. 2). This allowed memory to be assessed not in a binary fashion—remember or not—but in a parametric, continuous manner (Ma et al. 2014).

There were 4 experimental conditions intermixed within the experimental task (Fig. 2):

- Ignore condition: distracter stimuli were presented while maintaining information.
- Maintain (T1) the temporal control condition for ignoring—simple maintenance for the same duration.
- Update condition: a new pair of stimuli were presented during maintenance which replaced the previous memoranda as items that had to be maintained.
- Maintain (T2) temporal control for updating—simple maintenance for same the duration as the updated material.

All conditions shared the following common features. Participants saw 2 differently colored arrows (randomly orientated) presented at different spatial locations equidistant from screen center for 2 s. At the end of each trial, in the probe phase, they were shown an arrow, with a randomly offset orientation, and asked to rotate it so that it matched the original orientation in which it was presented. Both the ignore and update conditions involved dealing with irrelevant information—the former in a proactive manner and the latter in a retrospective fashion. The temporal controls for each condition served as simple maintenance conditions where participants stored and maintained information without having to deal with any irrelevant information. The cognitive requirements of the 4 different conditions were not explicitly communicated to participants. They were simply told that they had to remember only the arrows that were last presented with the letter “T”, which if displayed always appeared at screen center. The “T” stood for “target” and simply acted as a cue to instruct participants that they should remember the arrows displayed on that screen.

In the “*ignore condition*”, a pair of arrows was first presented for 2 s with a “T” at screen center, indicating that participants should remember these items. After a 2 s blank-screen delay, they were presented with another pair of arrows (again for 2 s), of different color and orientation to those shown previously. This second pair of arrows were distracters and had to be ignored, signaled by the fact that there was simply a fixation cross at screen center. Participants were then probed by being asked to reproduce the orientation of one of the target arrows, indicated by color, which was randomly drawn from the first pair of arrows seen on that trial. Thus, there was a 6 s delay between seeing target items and being probed on them.

In contrast, in the “update condition”, participants were presented with 2 sequentially presented pairs of arrows, both of which had to be successively encoded as targets, i.e., the letter “T” was presented on both frames. In this case, the probed arrow came from the second (most recently seen) pair of arrows, and the first frame was rendered irrelevant by the appearance of the second “T”. Thus, there was only a 2 s delay between seeing the target item and being probed on it.

Given that in the ignore and update conditions participants are probed on information retained for different periods, any difference in performance we observed between these 2 conditions could simply be due to the time difference. In order to rule this out, we had 2 control conditions that were calibrated to have exactly the same time retention period as there were for the target arrows in the ignore and update conditions. Thus, there was one maintain condition that served as the temporal control for the ignore trials (Maintain (T1) in Fig. 2), which was exactly the same as the ignore condition, except that no distracters were presented. Another maintain condition (Maintain (T2) in Fig. 2) acted as the control for the update condition and was exactly the same as that condition except that no information was presented in the first frame. These controls are important to exclude any effects due to simple temporal decay on WM, rather than ignoring or updating. The ignoring and updating trials were identical in every single way, except for presentation of the “T” on the first or second stimuli presentation (Fig. 2).

Patients were tested on 2 separate occasions—once “ON” their medication and once “OFF” their medication, with the order counterbalanced across patients. These 2 sessions were completed within a space of between 1 to 4 weeks. To facilitate a comparison with patients, healthy older adults were also tested twice. For PD patients, the task was shortened by 50%, so that the experiment contained 128 trials, 32 trials in each condition. Our comparison between patients and controls compensated for this fact (see below).

### Analysis

The main metric of performance used in this experiment was angular error, calculated as the absolute angular difference between the target orientation (the orientation of the probed bar) and the response orientation. All the data were analyzed with MATLAB 2015a and the statistical toolbox (The MathWorks, Inc.) using a hierarchical general linear mixed model as instantiated in the “fitlme.m” function.

Two models were evaluated to answer our key questions:

- First, we assessed the effect of the disease processes itself on memory by comparing the performance of patients (collapsed across medication session) to healthy older adults.
- Second, we examined the effect that dopaminergic medication has in PD patients on the ability to ignore and update information in WM.

In both models, retention interval (2 vs. 6 s), the presence of irrelevant information (ignore/update vs. maintain trials) and their interactions were entered as fixed effects. Subject was always entered as a random effect and any other random effects that warranted inclusion (improved model fit; lower Akaike information criteria (AIC) value) and their interactions were also entered as random effects. Note, however, that the significance of the effects presented here did not depend upon the inclusion of certain random effects.

The first and second models differed in that they included, respectively, disease and drug. For practical reasons, patients only performed half as many trials as the healthy older adults on each session. To make sure the number of trials being compared between patients and controls were equal, we only compared the performance of the patients to the first half of each of the sessions that the elderly controls performed. In all above models, maximum-likelihood was used to estimate model parameters. *F*-tests were used to examine the statistical significance of the fixed effects variables. Denominator degrees of freedom were estimated using a Satterthwaite correction and rounded to nearest integer.

It should be noted that in the above models a differential effect of ignore and updating, after correcting for time, would manifest as a significant interaction between retention interval (short and long) versus the presence of irrelevant information (maintain only or irrelevant information present).

### Modeling

In addition to angular error we can obtain additional clues about the underlying mental representations that support WM by examining changes in the pattern of errors according to disease and medication. One such model (Bays et al. 2009) has previously been used to uncover the unique pattern of errors associated with PD (Zokaei et al. 2014). The following mixture model was used to identify four sources of recall error.

$$p\left(\hat{\theta }\right)=\;\alpha \phi_{\kappa }\left(\hat{\theta }-\theta \right)+\;\beta \frac{1}{m}\overset{m}{\underset{i}{\sum }}\phi_{\kappa }\left(\hat{\theta }-\varphi_{i}\right)+\;\gamma \frac{1}{2\pi }$$

1. Variability in response (referred to as *kappa* or *κ*; Fig. 1*C*).
2. Probability of responding to the target orientation (α; Fig. 1*C*).
3. Probability of responding to nontargets (β; Fig. 1*E*).
4. Probability of guessing (γ; Fig. 1*F*).

Expectation maximization measures were used to derive the maximum-likelihood-derived (Myung 2003) parameters of κ, α, β, and γ (see Bays et al. 2009 for more details). Note, however, that three parameters (α, β, and γ) are not independent as they must sum to 1. In all conditions, orientations of items that were presented but not probed were entered into the model as nontargets (items that participants could misbind to). Thus, the other target item that had to be remembered—but was not probed—was entered as nontarget for all conditions. In addition, the irrelevant pair of items in the ignore and update conditions were entered as nontargets. Furthermore, to ensure that there are sufficient number of trials (*n* > 50) for a good model fit, we collapsed across drug sessions (ON vs. OFF) when comparing patients with controls on all 4 conditions (ignore, maintain (T1), update and maintain (T2)) and across conditions with (ignore and update) and without (maintain only) irrelevant information when comparing the effects of medication. Information on model fits is presented in the Supplementary Material.

Modeling is potentially important when it comes to adjudicating between whether forgetting in PD and the effects of dopaminergic modulation are due to decay or interference. If dissociable effects of retention interval and introducing irrelevant information are found on the various modeling parameters then this will give us important clues about whether forgetting occurs due to decay or interference. In the former case, it could be expected that the kappa (variability of precision) of recall is affected as a function of retention period as information is progressively degraded. In contrast, if forgetting is primarily due to interference-based processes, i.e., competition, then variable performance in the interference condition (ignore and update) should lead to differences in the probability of responding to the target. However, a problem with showing that declines in recall with retention period is due to decay is that irrelevant information may be spontaneously recalled. If this is the case then declines in recall due to retention period will be due to interference and not just decay.

One way to examine this issue is to look at the effects on recall when participants are actually presented with irrelevant information. This occurs in the ignore and update conditions and therefore provides a direct measure of the actual consequences of presenting irrelevant information. Arguably, this should be the same as spontaneously retrieving irrelevant information. Thus, if modeling reveals different effects of retention period and introducing irrelevant information on mental representations, this would potentially make it possible to separate out the processes of decay-based versus interference-based forgetting.

## Results

Four primary analyses are performed. First, we present overall performance differences between patients and controls, followed by modeling parameters to estimate the sources of error for the group differences that were found. Then, overall performance differences between patients ON and OFF their medication are analyzed, followed by modeling of the sources of error for the drug effects.

### Recall Error: Impaired According to Retention Period in PD

**Figure 3.**
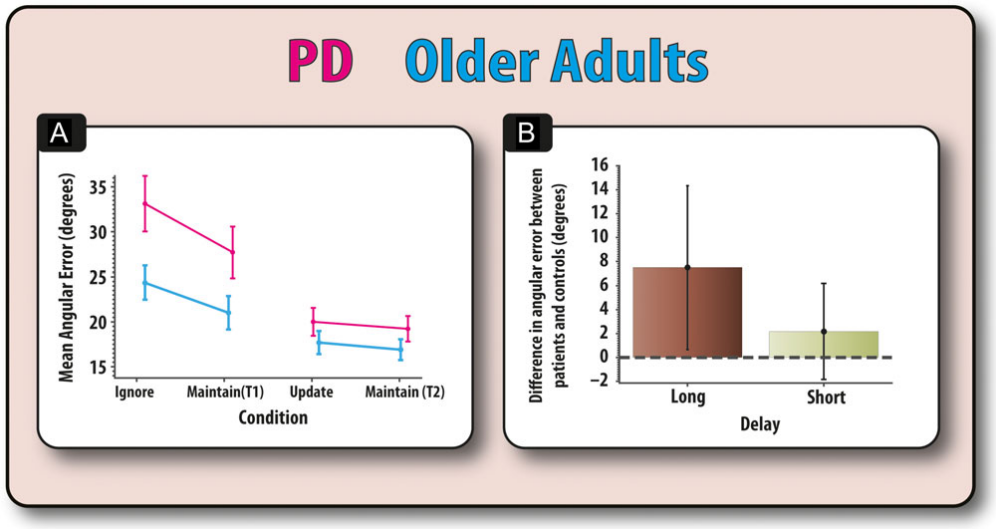
Performance of PD patients compared to older controls. (*A*) Mean angular error of patients and controls across all four task conditions. (*B*) Difference in angular error between patients and controls for the long retention trials (ignore and maintain (T1)) and short retention trials (update and maintain (T2)). Patients were disproportionately impaired at the long compared to the short duration trials. Error bars reflect the standard error of the mean.

We examined the effects of PD on recall performance (Fig. 3*A*) collapsed across ON and OFF sessions, compared to healthy controls (collapsed across Sessions 1 and 2). This analysis included disease, retention period (short vs. long), and the presence of irrelevant information (maintain only or with irrelevant information) as fixed factors, and subject as a random factor within disease. Overall recall error—angular error in reproducing the correct orientation of the target—was significantly higher on trials that contained irrelevant information (i.e., which required updating or ignoring) compared to simple, control maintain trials (*F*_1,36_ = 18.41, *P* < 0.001). Longer retention periods were also associated with impaired recall (*F*_1,36_ = 35.93, *P* < 0.001). There was a significant interaction between these 2 factors (*F*_1,36_ = 5.87, *P* = 0.002). This interaction was due to recall error on ignore trials being significantly higher compared to its temporal control (*F*_1,36_ = 19.80, *P* < 0.001), but this was not the case for update trials compared to its temporal control (*F*_1,36_ = 1.26, *P* = 0.27).

With regard to differences between groups, patients had lower performance (higher angular error) compared with controls (*F*_1,36_ = 4.32, *P* = 0.045). PD significantly affected the influence of retention period (*F*_1,36_ = 5.87, *P* = 0.02; Fig. 3*B*), such that patients were only significantly impaired on long (*F*_1,36_ = 5.45, *P* = 0.025), but not short retention periods (*F*_1,36_ = 1.67, *P* = 0.20). In contrast, disease did not significantly influence the effect irrelevant information (updating or ignoring) had on recall, neither was there a 3-way interaction between disease, presence of irrelevant information and retention interval (*F*s < 1). The increased susceptibility of patients to the effects of retention period remained the same irrespective of whether elderly controls were compared with patients ON or OFF medication (see Supplementary Materials). Similarly, there was no differential effect of session between patients and controls (Supplementary Materials).

### Modeling: Reduced Precision for Longer Delays in PD

Next, we examined how model parameters were affected by PD versus older controls.

#### Kappa

**Figure 4.**
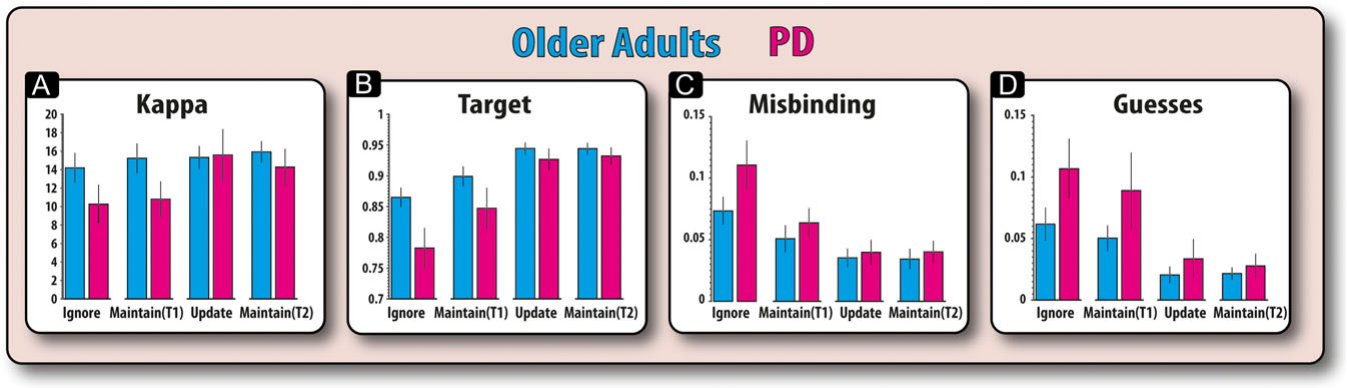
Modeling results for patients and controls (both sessions). (*A*) Disease was found to significantly affect kappa values according to retention period. PD patients showed a reduced kappa for retaining items for long but not short periods. (*B*) Disease did not significantly affect the probability of responding to the target according to delay (*C*) Similarly, disease did not affect misbinding according to delay (*D*) Also, disease did not significantly affect guess responses according to delay. Error bars reflect standard error of the mean (SEM).

This parameter refers to the concentration of responses around memoranda; a higher kappa indicates a higher concentration around presented items and is thus indicative of stored items having greater fidelity. We compared the Kappa for patients and controls according to time and the presence of irrelevant information in a mixed ANOVA (Fig. 4*A*). Having to hold information for long compared with short periods was associated with a significant reduction in kappa (*F*_1,34_ = 14.77, *P* = 0.001), an effect that was significantly modulated by disease (*F*_1,34_ = 7.62, *P* = 0.009). This interaction was due to patients having significantly reduced kappa, i.e., greater variability, for longer versus shorter trials (*t*(34) = 4.80, *P* < 0.001), but there was no such effect in controls (*t* < 1). Comparing the groups, PD patients did not have significantly lower kappa values compared to controls for long (*t*(34) = 1.71, *P* = 0.10) or short duration trials (*t* < 1). There was no significant interaction between time and presence of irrelevant information (*F* < 1). There was no main effect of disease and it did not significantly interact with any other variable and no other effects were significant (*F* < 1). It should be noted that equivalent nonparametric tests produced the same results as the parametric analyses. Wilcoxon signed-rank tests revealed that patients had significantly higher kappa values for longer, compared with shorter, duration trials (*Z* = 3.26, *P* = 0.001). However, there was no such significant effect of duration in controls (*Z* = 1.39, *P* = 0.163). A between-subject comparison of the effect of duration on kappa values for patients and controls (Mann–Whitney U), also revealed a significantly greater effect of duration (long minus short) on kappa values for patients compared to controls (*Z* = 2.61, *P* = 0.009).

#### Other Parameters in the Model

There were no effects of PD on the other model parameters representing the probability of responding to the target, misbinding or guessing (Fig. 4; Supplementary Materials).

In summary, these results suggest that the differential cost of time on WM fidelity in PD versus controls is due to changes in the underlying concentration of responses around memoranda (kappa). In patients, the precision of information decayed with longer maintenance durations on our task, whereas this effect was absent in older adults. Importantly, there were no significant differences between PD patients and controls for updating or ignoring information.

### Dopamine has Specific Positive Effects on WM Performance

**Figure 5.**
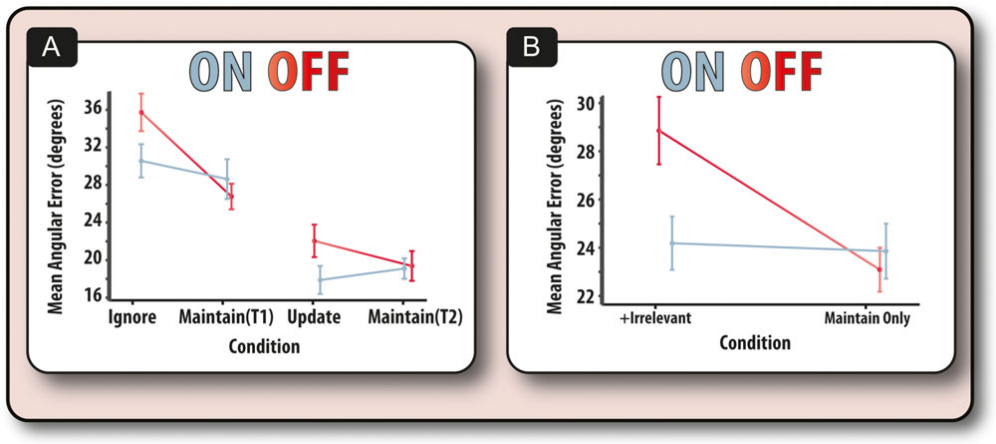
Performance for PD patients ON and OFF dopaminergic medication. (*A*) Mean angular error for patients ON and OFF their medication on all four tasks. Long duration retention involved ignore and its maintain control while short retention was required in the update condition and its maintain control. (*B*) Mean angular error of trials in which information only had to be maintained (T1 and T2) compared to trials in which irrelevant information had to be dealt with actively (ignore and update).

How does dopamine affect WM performance? Does it have its effect on updating, ignoring or only on maintenance duration? In PD patients, we examined within an ANOVA the effects of drug state (ON vs. OFF), temporal retention period and the presence of irrelevant information (ignore/update vs. simple maintain). Medication did not have a main effect on raw recall error (*F*_1,20_ = 1.27, *P* = 0.27). Crucially, however, dopamine affected performance according to the presence or absence of irrelevant information (*F*_1,28_ = 6.13, *P* = 0.019; Fig. 5*A*,*B*). Specifically, dopamine improved performance on trials that contained irrelevant information, i.e., both the ignore and update trials (*F*_1,19_ = 4.41, *P* = 0.049). Importantly, being ON, compared to OFF, dopamine had no effect on performance on the trials in which information only had to be retained (*F* < 1). We also examined the correlation between drug-induced improvement on ignoring (minus its control) and drug-induced improvement on updating (minus its control). This revealed that, across patients, there was no significant relationship between the 2 drug effects (*rho* = 0.275, *P* = 0.252).

As noted previously, performance was significantly affected by retention period (*F*_1,19_ = 32.18, *P* < 0.001), but crucially this was not modulated by drug (*F* < 1). Conditions with irrelevant information (ignore and update) were associated with worse performance than simple maintain trials (*F*_1,35_ = 6.94, *P* = 0.012). Drug did not significantly affect the interaction between irrelevant information and retention period (*P* = 0.32). Neither UPDRS nor equivalent l-dopa dose modulated the above effects (Supplementary Materials).

### Modeling: Dopamine Protects WM from Irrelevant Information by Reducing Guessing

**Figure 6.**
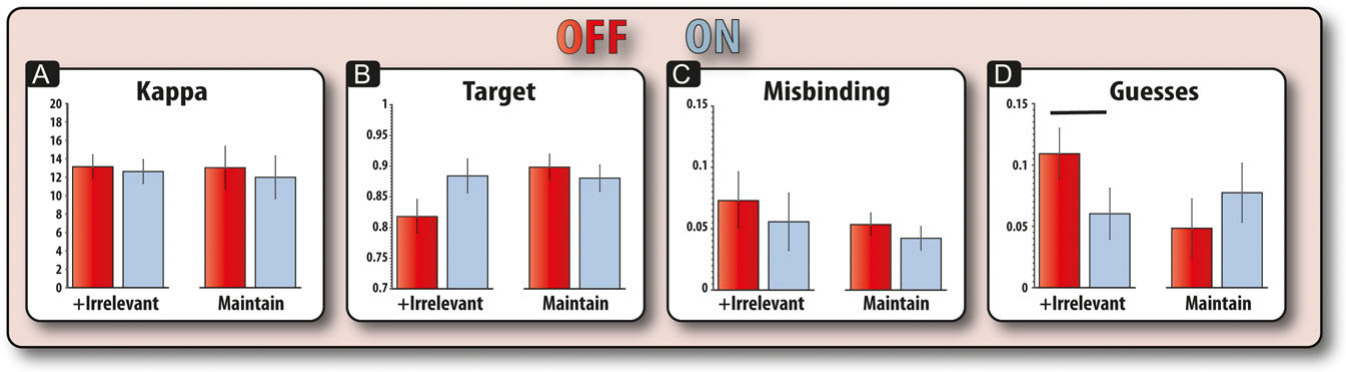
Modeling results for patients ON and OFF their medication. (*A*) Medication did not significantly affect kappa values. (*B*) Drug significantly modulated the probability of responding to the target, but only for trials that contained irrelevant information and not for the maintain only trials. (*C*) Drug did not significantly affect misbinding. (*D*) Drug did significantly modulate the probability of making a guess response. Drug reduced the probability of guess responses in trials that contained irrelevant information, but did not significantly affect guess responses on maintain trials. Error bars reflect the standard error of the difference (SED) between ON and OFF sessions.

Next, we sought to dissect out the sources of error that patients made using model parameters. We investigated the effect that drug had on conditions with irrelevant information (ignore and update) and conditions without irrelevant information (maintain only). Thus, to ensure we had sufficient numbers of trials, we collapsed across conditions, comparing trials with irrelevant information to maintain only trials. For kappa, the concentration parameter, there was no significant main effect of drug, presence of irrelevant information or interaction between drug and the presence of irrelevant information (*F*s < 1; Fig. 6*A*). Thus, the effect of drug on raw recall error (Fig. 5*A*) could not be due to changes in kappa (variability of response).

For the probability of responding to targets (Fig. 6*B*), there was again no significant main effect of drug (*F*_1,18_ = 0.289, *P* = 0.29), but irrelevant information did significantly affect this parameter. Irrelevant information (update and ignore trials) significantly diminished the probability of responding to the target orientation (*F*_1,18_ = 5.20, *P* = 0.035); and this was significantly modulated by drug (*F*_1,18_ = 7.80, *P* = 0.012). This was due to drug increasing the chances of responding to the target orientation when there was irrelevant information (*t*(18) = 2.25, *P* = 0.037), but not for the maintain only trials (*t*(18) = 0.93, *P* = 0.36).

The reduction in raw error (Fig. 5*A*) could have been driven by the two other sources of error—either participants could be responding to one of the nontarget orientations or they could be guessing. However, drug did not significantly affect misbinding (*F* < 1) nor interact with the presence of irrelevant information to influence misbinding (*F* < 1; Fig. 6*C*). The presence of irrelevant information also did not significantly affect misbinding (*F*_1,18_ = 2.84, *P* = 0.10). Thus, an increase in misbinding is not the source of the reduction in the probability of responding to the target.

For probability of guessing (Fig. 6*D*), there was no significant main effect of drug (*F* < 1) or irrelevant information (*F*_1,18_ = 1.94, *P* = 0.17). However, there was a significant interaction between drug and the presence of irrelevant information (*F*_1,18_ = 4.93, *P* = 0.039). This was due to drug decreasing guessing in the presence of irrelevant information (*t*(18) = 2.32, *P* = 0.032), but not on maintain trials (*t*(18) = 1.11, *P* = 0.280). Thus, the beneficial effect of medication in resisting irrelevant information observed in raw error of response (Fig. 5*A*) appeared to be mediated by a reduction in the amount of guess responses. Nonparametric tests (Wilcoxon signed-ranks) corroborated the above results. Patients OFF their medication made significantly more guess responses than patients ON their medication on trials that contained irrelevant information (*Z* = 2.15, *P* = 0.031), but not on maintain only trials (*Z* = 0.67, *P* = 0.50). This difference in the effect of dopaminergic medication on each condition was also significant (medication effect on trials with irrelevant information compared to the medication effect of maintain only trials (*Z* = 2.11, *P* = 0.035)).

To summarize, dopamine modified the probability of guessing but not kappa, only on manipulation trials in which irrelevant information (to be ignored or updated) was presented. This effect of being ON versus OFF dopaminergic medication contrasts sharply with the distinct differences in kappa between PD patients and controls observed only for longer retention periods, but not on irrelevant information trials.

## Discussion

In this study, we used a novel design to probe WM performance when people either have to ignore irrelevant information; update the contents of WM with new items so old ones are jettisoned and now become irrelevant; or simply maintain information (Fig. 2). We were able to dissociate the effects of medication and disease. PD patients' dopaminergic medication selectively improved their ability to accurately recall information in the presence of irrelevant information, irrespective of whether this information had to be ignored or updated (Fig. 5*A*). In contrast, their dopaminergic status did not have any effect on the ability to effectively maintain information across different retention periods. Intriguingly, it was the nondopaminergic element—increased susceptibility to temporal decay—that distinguished patients from healthy older adults (Fig. 3). Thus, the results of this study suggest there might be different components underlying the WM impairment in PD which, importantly, are modulated differentially by dopamine and the nondopaminergic pathology present in PD.

By applying a computational model of WM (Bays et al. 2009) we were further able to dissect out the underlying mechanisms behind the potential dopaminergic and nondopaminergic deficits and provide an indication about whether these changes are due to decay (decrements in fidelity) or interference-based processes. Differences between patients and controls were found to correspond to reductions in the precision of mental representations (indexed by the concentration parameter, kappa) with prolonged retention periods (Fig. 4*A*). However, comparison of PD patients (over both sessions) to controls showed no significant difference in the effect irrelevant information had on the precision of mental representations, either in the ignore or update conditions (Figs 3 and 4). Thus, independent of dopaminergic state, forgetting occurs in PD because information decays more quickly over time and therefore the basic process of maintaining information over time is impaired in patients compared to older controls.

In contrast to the general effects of PD, dopaminergic state (ON or OFF) altered the likelihood of WM recall being corrupted by guessing when irrelevant information had to be suppressed (Fig. 6). In addition, neither a change in dopaminergic state nor the introduction of irrelevant information led to a change in the fidelity of information (kappa; Fig. 6*A*). This suggests that neither the introduction of irrelevant information nor dopaminergic medication impact upon recall through modifying the precision of mental representations. Rather, dopaminergic medication in PD serves to guard against relevant information being disrupted by irrelevant information. These results suggest that both decay and interference-based processes exert dissociable effects on WM recall, which might rely on different neural substrates or mechanisms.

### Forgetting in PD: The Effects of Temporal Decay

There is an active literature that seeks to understand whether forgetting in healthy humans is due to decay or interference-based processes (Portrat et al. 2008; Farrell et al. 2016). One school of thought, crystallized in the task-based resource sharing (TBRS) model, conceptualizes the vivacity of memoranda to be governed by the process of temporal decay, unless actively refreshed (Barrouillet et al. 2012). Alternatively, other models have proposed that there is a very limited effect of time on the quality of memoranda unless there is some distracter or irrelevant information to deal with (Lewandowsky et al. 2010). We observed in older adults as well as PD patients (Fig. 3*A*) that information can be lost by extending the retention period from just 2–6 s, independently of whether irrelevant information is present. Thus, the data presented here provide support for the contention that time, on its own, can play an important role in the quality of recall.

We were able to evaluate which of these putative mechanisms—decay or interference—determines the lifespan of memoranda in PD. The results crucially suggest that both factors contribute, but that different processes—potentially dopaminergic and nondopaminergic—may be responsible for interference and decay-based forgetting respectively. The results from our model-based analysis of WM help us to further understand the mechanisms through which information decays at a quicker rate in PD, irrespective of dopaminergic status (Fig. 4*B*).

The source of patients' recall errors was predominantly found to be a reduction in memory precision over longer retention periods (Fig. 3*A*). One potential source of this deficit is changes in the oscillatory brain dynamics that are necessary for successful WM. The precision of memoranda, but not the guess rate, has been found to relate to changes in the power of neuronal oscillations in the alpha (~10 Hz) band in posterior cortical regions (Myers et al. 2014; Poliakov et al. 2014) and changes in alpha power are thought to index changes in the functional inhibition of irrelevant cortical areas (Jensen and Mazaheri 2010) or controlled access to maintained information (Klimesch 2012). Future studies might profitably examine whether these disturbed dynamics underlie the increased temporal decay in WM in PD patients.

The neurochemical basis of this component of the WM deficit may partially reside in the nondopaminergic pathologies present in PD, such as those involving noradrenaline (Zarow et al. 2003), serotonin (Kish et al. 2008), GABA (Emir et al. 2012), and acetylcholine (Dubois et al. 1983), which may be responsible, either singly or in combination, for patients’ impaired recall over longer durations. Each of these abnormalities has been hypothesized to lead to cognitive deficits in PD patients (e.g., Calabresi et al. 2006; Kehagia et al. 2010). Observations on the effects of drugs that selectively alter each of these neurotransmitters would therefore be important to determine whether they contribute to accelerated decay of information over time in PD.

### Dopamine Creates Memories that are Robust to Irrelevant Information

Previous investigations have produced diverging results on the precise nature of dopamine’s effect on WM in PD (Poewe et al. 1991; Fournet et al. 2000; Lewis et al. 2005; Cools et al. 2010). The results of the current study provide some clarity on this issue. Here, replenishing dopamine in PD patients was found to selectively improve WM recall when irrelevant information had to be dealt with—irrespective of whether that information had to be ignored or updated. In contrast, dopamine had no effect on the recall of information according to the temporal duration with which it needed to be retained. This suggests that dopamine contributes to the executive component of WM that deals with the organization of relevant and irrelevant information in WM, consistent with some previous findings (Dalrymple-Alford et al. 1994; Gruszka et al. 2016). This conclusion differs from a previous study that also used a precision-based method of assessing WM and found that medication improved overall recall in PD patients (Zokaei et al. 2015). However, there are important methodological differences between that study and the present one. In addition to differences in task structure (timing and number of items), the previous study only examined WM in patients before and after initiating dopaminergic medication. Thus, one interpretation of the Zokaei et al. (2015) results is that they were driven by session effects (testing patients OFF their medication always preceded testing patients ON their medication). Given that we counter-balance the order of drug withdrawal, this criticism is not applicable to the current study.

In one influential model of the role of dopamine in WM, a division of labor is proposed between the direct D_1_ receptor-dominated “Go” pathway and the indirect D_2_ receptor-dominated “NoGo” pathway that link the striatum to the cortex, with the former promoting and the latter preventing the entry of information into WM (Hazy et al. 2007). Activation of the Go pathway is thought to reinforce the representation of relevant information, whereas NoGo pathway activation should diminish the representation of irrelevant information (Moustafa et al. 2008), i.e., the Go pathway opens the gate to WM whereas the NoGo pathway closes it.

A classic model of the pathophysiology of PD (DeLong 1990) proposes that there is an imbalance between the Go and NoGo pathway, and that dopaminergic medication exerts its therapeutic effects by enhancing Go activity but reducing NoGo activity. In the mnemonic domain, this should correspond to potentially improved updating but impaired ignoring (Wiecki and Frank 2010). Thus, updating should be selectively improved by medication, but distracter-resistance should be impaired. This was not observed in our study. In fact, we obtained positive evidence that replenishment of dopamine in PD patients improves both ignoring and updating.

Moreover, we found that there was no significant correlation between the dopaminergic-induced benefit on ignoring and the dopaminergic-induced benefit on updating, suggesting that there may not be a common neurophysiological substrate that is promoting a trade-off between cognitive stability and cognitive flexibility (Hazy et al. 2007; Durstewitz and Seamans 2008)—at least within the manner and modality tested here. This suggests that dopamine influences ignoring and updating through separate neurocognitive mechanisms. A conclusion which is consistent with there being separate neural substrates for distracter-resistance and updating or cognitive flexibility (Cools and D’Esposito 2011; Fallon and Cools 2014; Fallon et al. 2016a), but suggests that a trade-off between the two is not obligatory. Similarly, recent models and empirical data (Chatham et al. 2014; Chatham and Badre 2015) have argued for a separation between input gating and outputting, with the former responsible for making sure that irrelevant items do not enter WM. By contrast, the output gating function would act to prevent now nonrelevant items from being able to affect behavior. It is possible that our assay of ignoring is closely aligned with input gating (filtering out distracters), whereas updating is more reliant upon output gating (so that old items are jettisoned and do not interfere). Thus, withdrawing patients from their medication impairs both of these functions. Future studies, using more pharmacologically specific interventions, should seek to decompose these effects. For example, examining the effect of selective D_2_ dopamine receptor stimulation.

In any case, by using a modeling approach, we were able to show that dopamine influences recall in trials in which irrelevant information is present by decreasing the probability of guess responses (Fig. 6*D*). Guess responses occur when participants' responses bear no relationship to the orientations of the targets or the nontargets (Fig. 1*F*). The increase in guesses in the presence of irrelevant information in the OFF state suggests that patients were, on some trials, more likely to be completely amnestic for the relevant mental representation. In other words, it was not the case that WM representations were simply less vivid and noisier after irrelevant information, but rather that the relevant information was no longer effectively represented.

### Possible Alternative Accounts

An alternative explanation for the dopaminergic effects observed here is that, rather than reflecting how patients dealt with irrelevant information, they occur because there are more items in the ignore and update conditions than in the maintain conditions (4 vs. 2 items). A corollary of this interpretation is that patients were treating the ignore and update trials as 4-item conditions and thus performance on the ignore and update trials should be identical. However, PD patients, irrespective of medication status, performed better on the update than on ignore trials (Fig. 3*A*), which would not be expected if they were both being treated equivalently as four-item trials. Similarly, if the update condition was being treated as a 4-item trial, it seems unlikely that patients would be performing better on this condition than on the long retention 2-item maintain task. Thus, there is little evidence to suggest that the observed difference in ignore and update performance as a function of medication was due to load.

Another alternative explanation of the results is that they are due to level of difficulty. For example, it might be argued that patients have accelerated temporal decay on the long duration trials because they were more difficult or more effortful. Indeed, it is true that across all of the different samples we tested, trials with longer retention intervals were harder (associated with increased recall error) than the short retention intervals. Thus, this result could also be incorporated into a framework where the neural degeneration present in PD makes them less able to perform effortful tasks through being unable to pay the intrinsic control costs required by these actions (Manohar et al. 2015). However, the same could not be said for our dopaminergic effects. Here, dopamine improved performance on tasks in which irrelevant information had to be dealt with (ignoring and updating) but had no effect in modulating the effect of temporal duration, i.e., effort on recall. Thus, while the disease-specific effects may be due to changes in the ability to exert cognitive effort, the same cannot be said for the dopaminergic effects reported here. In any event, the decay effects seem dissociable from the mechanisms underlying the effects on ignoring and updating.

Finally, it could be argued that the demonstration that the reduction in precision with increasing retention period in patients does not necessarily reflect decay, but could also occur due to an interference-based process. That is, prolonging the retention period increased the probability that patients will spontaneously retrieve irrelevant information, thereby decreasing the precision of the relevant information. While such a mechanism can arise in the early phase of retention (Pertzov et al. 2016), several aspects of the present results suggest that this is unlikely to explain forgetting in this instance. Specifically, introducing irrelevant information (in the ignore and update conditions) did not affect the precision of memoranda, but did affect the rates of misbinding and guessing (Fig. 4*A*–*D*). Thus, actually introducing irrelevant information itself did not change precision. This finding makes it very unlikely that the forgetting effects due to increasing retention period were actually due to patients retrieving irrelevant information during the delay period. This also aligns with computational models of WM, which demonstrate that interference from irrelevant items in memory would disrupt whole items reducing the probability of recalling them, whereas time primarily causes drift in representations, reducing precision or fidelity (Wei et al. 2012).

One caveat that could possibly be added to our conclusions is that, due to insufficient trials, we were unable to separately examine how medication in patients differentially affected retention over time versus resilience to irrelevant information. Therefore, medication may have had separate effects on the modeling parameters according to delay. However, it should be noted when analyzing overall WM recall error, that medication did not affect performance according to delay period. Thus, there is little evidence to suggest that medication would affect the modeling parameters according to delay.

### Implications

The above caveats notwithstanding, the results of this study suggest that there are limits to the extent to which dopamine can be used to ameliorate cognitive deficit in PD. Recent considerations suggest two broad mechanisms might contribute: a frontal dysexecutive syndrome that might be modulated by dopamine and a posterior cortical syndrome that is not altered by dopamine (Kehagia et al. 2012; Gratwicke et al. 2015). Our findings might be considered to be consistent with this general proposal, with dopamine affecting the ability to deal with irrelevant information but PD also being associated with potentially nondopaminergic mechanisms that lead to increased rates of information decay over time.

## Supplementary Material

Supplementary Data
