# Supplementary material for: Fractionating the Neurocognitive Mechanisms Underlying Working Memory: Independent Effects of Dopamine and Parkinson’s Disease
Source: Cereb Cortex. 2017 Oct 13;27(12):5727–38. doi: 10.1093/cercor/bhx242 (PMC5939219; doi:10.1093/cercor/bhx242)
Supplement: Supplementary Data [file bhx242_supplementary_files.zip › bhx242Supplemental_materials_2ndRound.pdf]

Supplemental Materials

	Patients	Controls
**Age**	65.7 (4.99)	68.47(3.82)
**ACE**	95.61 (5.00)	96.62 (3.20)
**Gender (male:female)**	10:9	9:8
**Equivalent l-dopa**	673.43 (373.4)	N/A
**UPDRS III motor score**	17.89 (9.50)	N/A

Supplementary Table 1. Patient and control demographics. [all p's > .05].

Model Evaluation

Model evaluation was done similarly to Fallon et al (2016), where we compare the model fits (Akaike Information Criterion; AIC) with and without the salient model parameters (misbinding and guessing).

We did this separately for our two main analyses (the patients and controls (collapsed across sessions; Figure 4 data) and for patients on their own (collapsed across tasks; data in Figure 6).

Firstly, for patients and controls, across all four conditions (ignore, maintain (t1), update and maintain (t2), we examined the AIC for three models: full model (all parameters), full model without the misbinding parameter and full model without the guessing parameter). As can be seen from S1, the full model was associated with better fit overall (lowest AIC value), and for both patients and controls.

Secondly, we compared patients ON and OFF medication (collapsed across conditions with irrelevant information (ignore and update) and both maintain conditions). Again, the full model produced the best fit (lowest AIC value), when patients were ON and OFF medication.

	Full model	Full model minus misbinding parameter	Full model minus guessing parameter
Patients	4804	4910	5028
Controls	4322	4451	4651
Overall	9126	9361	9679

Supplementary Table 2: Model fits (AIC values) for patients and controls (each condition modelled separately, collapsed across session)

	Full model	Full model minus misbinding parameter	Full model minus guessing parameter
Patients OFF	3439	3498	3848
Patients ON	3124	3165	3545

Supplementary Table 3: Model fits for patients ON and OFF medication (collapsed across conditions with irrelevant information (ignore and update) and both maintain conditions).

Effect of disease on model parameters

Probability of responding to target

For the probability of responding to the target orientation (**Figure 4B**), time ($F(1,34) = 36.38$, $p<0.001$) and presence of irrelevant information ($F(1,34)= 9.52$, $p=0.004$) decreased the propensity to respond to the target. Time and irrelevant information interacted significantly ($F(1,34)= 9.11$, $p=0.022$) with ignore trials having less responses to the relevant target orientation vs. their temporal control ($t(34)= 3.3$, $p<0.001$), but this was not the case for update vs. temporal control ($t<1$). There was no significant main effect of disease ($F(1,34) = 2.80$, $p=0.10$) or interaction between time and disease ($F(1,34)= 3.09$, $p=0.09$); none of the other effects were significant (p's>.32).

Misbinding

For misbinding (**Figure 4C**), again, both time ($F(1,34)= 12.35$, $p= 0.001$) and the presence of irrelevant information ($F(1,34)= 10.17$, $p=0.003$) significantly affected the level with which a non-target was reported. A significant interaction between time and presence of irrelevant information ($F(1,34)= 9.40$, $p = 0.004$) was due to increased misbinding occurring in the ignore trial compared to its temporal control ($t(34)= 4.63$, $p<0.001$), but not so for update ($t<1$). There was no significant main effect of disease ($F(1,34) = 1.50$, $p = .23$). Disease did not significantly interact with any other factor ($Fs<1$).

Random guessing

For the probability of guessing (**Figure 4D**), only retention period significantly affected this parameter ($F(1,34)= 20.60$, $p<0.001$), with longer durations being associated with increased guessing. There was no significant main effect of disease ($F(1,34)= 1.73$, $p=0.20$), interaction between disease and time

($F(1,34) = 2.36$, $p = 0.13$). There was also no effect of irrelevant information on guesses ($F(1,34) = 1.31$, $p = 0.26$). No other effects were significant (Fs<1).

Comparing patients on and off their medication to controls.

For the interested reader, we re-ran our analysis comparing patients and controls, but compared elderly controls to patients on and off their medication separately. Firstly, we compared only patients in their 'off' state to the healthy elderly control group. This analysis revealed that there was a main effect of disease group ($F(1,35) = 5.32$, $p = .027$), with patients making more errors compared to controls. Similar to comparing patients collapsed across drug state, there was a significant interaction between disease and the length of the retention period ($F(1,32) = 5.32$, $p = .027$). This was due to patients being significantly impaired at the long retention periods ($F(1,34) = 6.49$, p = .015), but not for short durations (F(1,34) = 2.45, p = .126). There was a significant interaction between disease and the presence of irrelevant information ($F(1,32) = 6.55$, $p = .015$). Patients OFF were not significantly impaired on maintain only trials (F(1,35) = 3.64, p = .064), but they were significantly impaired on the trials that contained irrelevant information (F(1,34) = 6.16, p = .018). There was no significant three-way interaction between disease, retention period and the presence of irrelevant information F(1,33) = 1.54, p = 22). Cumulatively, WM recall in patients OFF medication was impaired both by the need to maintain information for longer periods of time and the presence of irrelevant information. However, these two factors did not interact.

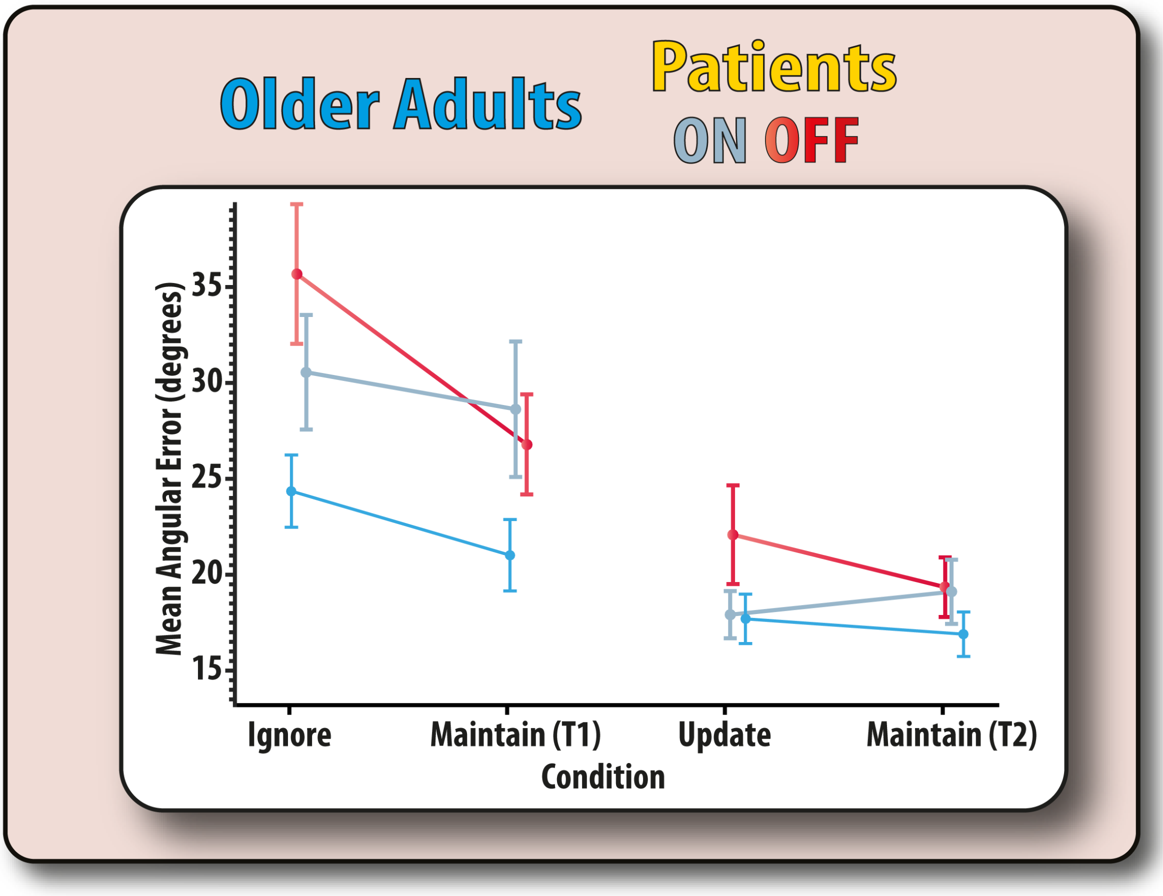

Supplemental Figure 1. Angular error across all four tasks for patients *in the OFF state*, *ON state* and healthy older adults. Error bars reflect the standard error of the mean.

Secondly, comparing the patients in the 'ON' state to the elderly control group, there was no main effect disease ($F(1,35) = 2.73$, $p = .010$). There was a significant interaction between disease and retention period ($F(1,33) = 6.87$, $p = .013$), with patients being impaired on the longer retention trials ($F(1,35) = 4.17$, $p = .048$) but not on short retention trials ($F<1$). There was no significant interaction between disease and the presence of irrelevant information ($F(1,33)= 1.51$, p .22) or three-way interaction between disease, retention period and presence of irrelevant information ($F<1$). Thus, even when patients were taking their medication, they were still disproportionately affected by retention period compared to the healthy older adults.

Session effects in patients and controls

As a control analysis, we examined whether healthy controls received a disproportionate advantage from performing more trials in the first session compared to patients. Recall error was significantly lower on the second compared to the first session ($F(1,36) = 20.72$, $p < .0001$). Patients had significantly higher rates of recall error compared to controls ($F(1,36) = 4.33$, $p = .044$). Crucially, however, there was no significant interaction between disease and session ($F(1,36) = 1.31$, $p = .26$). Thus, there is no evidence that healthy controls received a disproportionate benefit across sessions compared to patients.

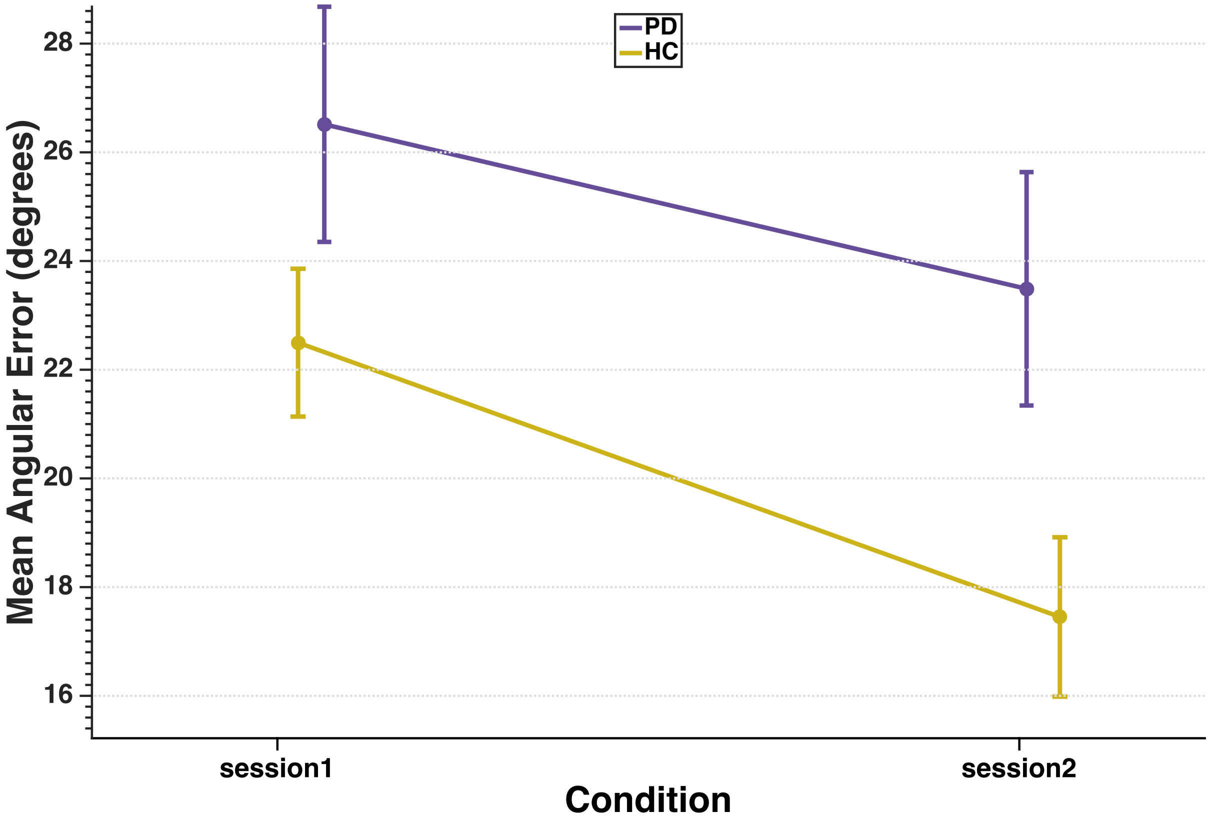

Supplementary Figure 2. Angular error across sessions PD patients and healthy older adults. Error bars reflect the standard error of the mean.

The role of clinical covariates in influences the effect drug has on working memory

Supplemental analyses examined the effect of, separately, putting equivalent levodopa dose and UPDRS motor score into the above analysis as z-scored covariates. The inclusion of UPDRS score did not alter the significant interaction between drug and presence of active condition ($F(1,25.41) = 6.69$, $p=0.015$), and there were no significant interaction effects between UPDRS score and the other experimental variables (ps>0.22). There was, however, a strong trend towards a main effect of motor score ($F(1,18) = 4.37$, $p=0.050$), with a higher motor score being associated with higher overall error. A similar analysis using equivalent l-dopa dose as a covariate (see method for calculation), found that inclusion of this covariate did not alter the significant of the interaction between drug and the presence of irrelevant information (F(1,25.3, 7.35, p = .011). There was no main effect of l-dopa dose or significant interaction with other variables (ps >0.23).
